# A Case of Uncomplicated Duodenal Diverticulosis Presenting With Right Upper Abdominal Pain

**DOI:** 10.7759/cureus.64062

**Published:** 2024-07-08

**Authors:** Lubna Saffarini, Yasmin H Kazim, Shaikh Sayeed Iqbal, Mahmoud Naji, Manar Butros

**Affiliations:** 1 Emergency Medicine, Rashid Hospital, Dubai, ARE; 2 Radiology, Rashid Hospital, Dubai, ARE

**Keywords:** small bowel diverticulosis, duodenal diverticulosis, acute cholecystitis, abdominal pain, duodenal diverticula

## Abstract

Small bowel diverticulosis is not a common disease entity; however, it is increasingly diagnosed and linked to various gastrointestinal complaints. Although rare, complications can occur and may sometimes require surgical or endoscopic intervention. Furthermore, suspecting and diagnosing duodenal diverticulosis (DD) can be challenging due to the variety of presenting signs and symptoms. Much of our current knowledge comes from case reports and series. This report aims to document a case of DD presenting with severe right upper quadrant pain mimicking the signs and symptoms of acute cholecystitis. It also reviews and summarizes the available literature on the clinical manifestations of DD, its diagnostic approach, treatment modalities, and possible complications encountered in the ED.

## Introduction

The duodenum is the second most common location for diverticular disease after the colon, and it is documented in up to 22% of the population [1,2]. There is no gender preference related to its occurrence, unlike the jejunum and ileum, where their incidence is 2.3% and they are more likely to occur in males [3]. Even though duodenal diverticulosis (DD) is mostly asymptomatic and a diagnosis is made incidentally, complications do occur [2]. Out of the 10% of symptomatic patients, only 1-2% will require surgical intervention [2].

Diverticula of the intestine can be divided into two categories: true or congenital and acquired [2,4]. True diverticula are due to a congenital anomaly occurring during weeks seven to 10 of gestation; they contain all three layers of the intestinal wall and are relatively less common than their acquired counterpart [2,4]. Congenital diverticula can be further subdivided into intraluminal and extraluminal [1]. Acquired or pseudo-diverticula occur due to a herniation through a focal defect in the bowel wall, which contains only two layers [2]. Ninety-five percent of these diverticula arise from the medial duodenal wall, and 62% of these arise from the second portion of the duodenum [2]. In some cases, they occur at the entry of the common bile duct, an entity termed juxtapapillary duodenal diverticula (JDD) [2].

We present the case of a 26-year-old female who presented with severe right upper quadrant pain, suggestive of acute cholecystitis. Upon evaluation, she was diagnosed with an uncomplicated DD.

## Case presentation

A 26-year-old female presented to the ED complaining of right upper quadrant abdominal pain. The pain had started a week before her hospital visit, radiating to her left upper back and shoulder and persistently worsening. It was also associated with fever and chills. She denied any history of vomiting, chest pain, urinary complaints, diarrhea, or constipation. On examination, she had a fever of 38.8 °C, right upper quadrant tenderness with a positive Murphy’s sign, and epigastric tenderness. Point of care ultrasound (POCUS) showed a distended gallbladder without pericholecystic fluid, stones, or wall thickening, and a cystic structure adjacent to the gallbladder was noted. Initial laboratory workup revealed a normal full blood count, liver function tests, bilirubin, lipase, urea and electrolytes (U&E), and creatinine, with mildly elevated CRP (25.7 mg/L).

**Figure 1:**
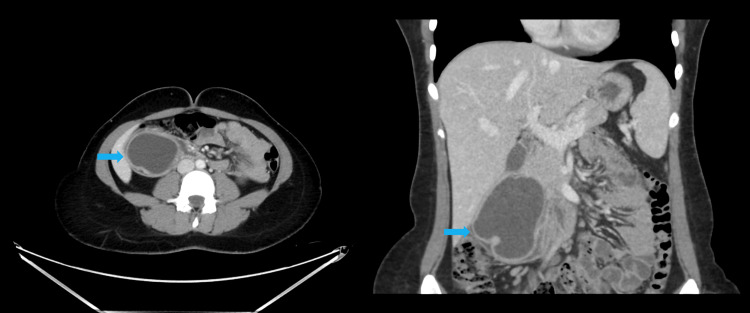
Axial (left) and coronal (right) portovenous phase of CT abdomen with IV contrast showing an 8.3 × 4.0 cm tubular cystic lesion (blue arrows) in the second part of the duodenum The change in the shape of the tubular structure is evident across three phases: portovenous, post-oral contrast, and lateral decubitus (Figures 1-3). These findings suggest a large intraluminal DD (blue arrows) in the second and third parts of the duodenum, without signs of cholecystitis, diverticulitis, or other complications. DD, duodenal diverticulosis

**Figure 2:**
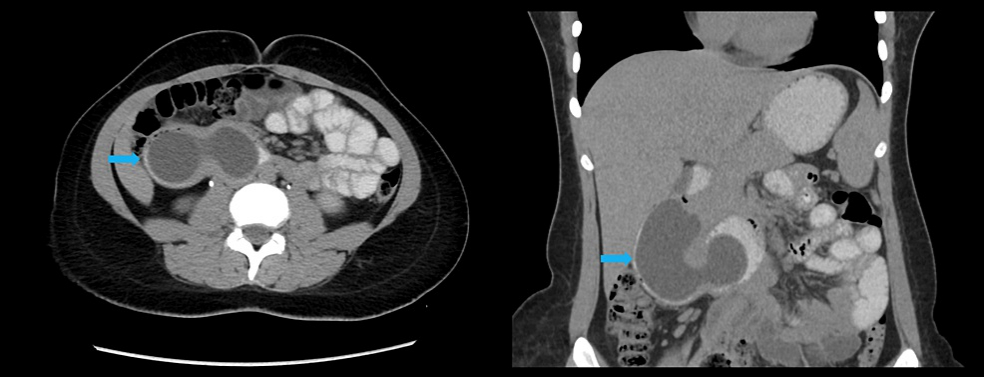
Axial (left) and coronal (right) post-oral contrast images of the CT abdomen showing an 8.3 × 4.0 cm tubular cystic lesion (blue arrows) in the second part of the duodenum The change in the shape of the tubular structure is evident across three phases: portovenous, post-oral contrast, and lateral decubitus (Figures 1-3). These findings suggest a large intraluminal DD (blue arrows) in the second and third parts of the duodenum, without signs of cholecystitis, diverticulitis, or other complications. DD, duodenal diverticulosis

**Figure 3:**
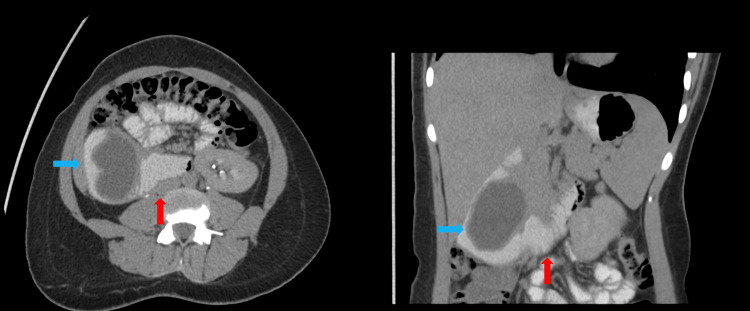
Axial (left) and coronal (right) right lateral decubitus images of CT abdomen with contrast showing an 8.3 × 4.0 cm tubular cystic lesion (blue arrows) in the second part of the duodenum extending into the proximal third of the duodenum (red arrows) The change in the shape of the tubular structure is evident across three phases: portovenous, post-oral contrast, and lateral decubitus (Figures 1-3). These findings suggest a large intraluminal DD (blue arrows) in the second and third parts of the duodenum, without signs of cholecystitis, diverticulitis, or other complications. DD, duodenal diverticulosis

Given the suspicion of acute cholecystitis based on history, examination, and POCUS findings, she underwent further imaging with CT of the abdomen with IV and oral contrast. This revealed a large tubular cystic structure measuring 8.3 × 4.0 cm, showing changes in shape with peristalsis extending into the lumen of the second to proximal third of the duodenum. These changes were appreciated in the portovenous phase (Figure 1), delayed post-oral contrast phase (Figure 2), and lateral decubitus scans (Figure 3). These findings were consistent with a large intraluminal DD in the second and third parts of the duodenum, without evidence of cholecystitis, diverticulitis, or other complications.

Her pain was managed effectively with an intramuscular injection of diclofenac sodium. After discussing the findings and the need for follow-up with gastroenterology and general surgery, she decided to return to her home country. She was discharged on empirical ciprofloxacin 500 mg twice daily for five days, pantoprazole 40 mg once daily, paracetamol one gram three times daily as needed, mefenamic acid three times daily as needed, and metoclopramide 10 mg three times daily as needed. Unfortunately, subsequent follow-up and outcomes of her treatment plan could not be obtained due to loss of contact with the patient.

## Discussion

After thoroughly reviewing the available literature, we found seven articles that matched our search criteria, including reviews, systematic reviews, randomized controlled trials, observational studies, meta-analyses, case reports, and clinical studies in English. We extrapolated two pictorial reviews [5,6], two case reports [7,8], and three reviews [2,3,9]. The articles in this review were cumulatively published in 2022, 2021, 2020, 2018, 2014, 2012, and 1999. To our knowledge, we could not find any articles published about the role of POCUS in diagnosing uncomplicated duodenal diverticula in the ED.

Diagnosis

CT imaging is the study of choice for diagnosing small bowel diverticula [3]. They are seen as saccular outpouchings or masses filled with air, fluid, an air-fluid level, contrast material, or debris adjacent to the normal duodenal lumen [2,6]. Periampullary diverticula may mimic a pancreatic or choledochal cyst when filled with fluid [6], and a DD filled with gas and debris may mimic a pancreatic head neoplasm [2]. To confirm the diagnosis of DD, direct luminal continuity with the duodenum should be seen [6]. Ultrasound imaging can also be utilized, despite its lower sensitivity [3]. The findings of DD on ultrasound have been described as a persistent bright linear or concave echo that obscures visualization of the normal pancreatic head [2].

Complications

It is noted that 90% of small bowel diverticula are asymptomatic; however, some complications can ensue, with mortality rates that can be as high as 50% [3]. Multiple possible complications have been documented in the literature associated with DD [2]. As with any diverticular disease, there is a risk of perforation with a mortality rate of 30% [2]. Duodenal diverticulitis is a complication that can be difficult to diagnose due to its presenting symptoms that mimic other complaints such as acute cholecystitis, pancreatitis, ulcerative disease, and colitis [2]. Another complication uncommonly encountered but documented in the literature is bleeding from the DD [2]. Patients usually present with hematemesis or melena, and the cause of the bleeding could be attributed to erosions, neoplasms, angiodysplasia, drug side effects, and ulcers [2].

JDD can cause biliary and pancreatic obstruction when the intraluminal pressure increases within the lumen, obstructing the ducts [2]. The term “Lemmel’s syndrome” is used when the JDD compressing the ducts causes obstructive jaundice [2]. Cholangitis can occur likewise [2]. Some papers have also documented that JDD is associated with biliary stones due to biliary stasis and bacterial contamination [2]. Acute pancreatitis is also another complication that is associated with both intraluminal and extraluminal diverticula [2].

One case report documented a case of congenital DD in a four-year-old child [7]. Even though the disease goes unrecognized clinically in most cases and symptoms are usually recorded in adulthood, the patient was incidentally diagnosed after ingestion of a foreign body that caused the diverticulum to rupture, and the patient presented with signs and symptoms of obstruction [7].

Management

Since DDs are more commonly asymptomatic, the treatment is usually conservative. However, complications occur at a rate of 10-20%, and further management depends on the type of complication [9]. The most common complication is duodenal diverticulitis [9], where the treatment plan includes an initial attempt at conservative management or endoscopic treatment [5]. If that fails, then surgery consisting of a transduodenal diverticulectomy is usually performed because of the risk of perforation, which carries a mortality rate of up to 30% [5]. There is no consensus management plan agreement in cases of ruptured DD. A systematic review of 47 cases stated that 34% of the patients were successfully managed by nonsurgical conservative observation, which is usually reserved for stable patients without signs of peritonitis [1]. Stable patients who failed to improve, patients with peritonitis, and unstable patients require surgical intervention where intraoperative assessment is required to determine the most appropriate surgical technique [1]. In cases of hemorrhage, an endoscopy is indicated unless there is severe bleeding where surgical intervention and resection are indicated [9]. Surgical resection is also advised in cases of intestinal obstruction due to multiple potential causes, such as a distended diverticulum, stricture or adhesion from prior diverticulitis, volvulus, or even nonmechanical obstruction due to dyskinesia [9]. When the DD is intraluminal, the obstruction can be managed endoscopically [9]. In the occurrence of Lemmel’s syndrome or obstructive jaundice, endoscopic extraction, extracorporeal shockwave lithotripsy, or surgery, such as a diverticulectomy or sphincterotomy with or without stent placement, may be indicated [8,9].

## Conclusions

Our patient presented with right upper abdominal pain and signs and symptoms of acute cholecystitis. She was suspected of having a cystic structure adjacent to the gallbladder on POCUS and was incidentally found to have uncomplicated DD without any complications or evidence of cholecystitis on CT imaging of her abdomen with IV and oral contrast. Unfortunately, she opted to follow up at another healthcare facility in her home country, so we do not have information on her prognosis. To our knowledge, there are no reports of cases being suspected or diagnosed by POCUS in the ED. Further research is needed to better understand the occurrence of DD and its presenting complaints, especially in the emergency setting where it is an unlikely cause of abdominal pain to be considered.
